# Ameliorative Effect of Dangguibuxue Decoction against Cyclophosphamide-Induced Heart Injury in Mice

**DOI:** 10.1155/2018/8503109

**Published:** 2018-11-01

**Authors:** Kun Liu, Xiu-mei Ren, Qing-sheng You, Ming-Ming Gu, Fei Wang, Shuo Wang, Chun-Hui Ma, Wei-Nan Li, Qing Ye

**Affiliations:** ^1^Department of Cardiothoracic Surgery, Affiliated Hospital of Nantong University, Nantong, China; ^2^Department of Traditional Chinese Medicine, Affiliated Hospital of Nantong University, Nantong, China; ^3^Department of Obstetrics and Gynecology, Affiliated Hospital of Nantong University, Nantong, China

## Abstract

Dangguibuxue decoction (DBD), a kind of Chinese herbal medicine, has been widely used to treat blood deficiency disease in China. In this experiment, we studied the effects of the Dangguibuxue decoction (DBD) on the myocardial injury induced by cyclophosphamide in mice. Alanine aminotransferase (ALT), aspartate aminotransferase (AST), creatine kinase (CK), and lactic dehydrogenase (LDH) in serum were detected by commercial kits. Total white blood cell (WBCs), platelets, and cytokines pathological changes of heart tissue were also examined. In addition, the protein levels of the NF-кB pathway were detected to reveal its mechanism. The results showed that DBD significantly decreased the levels of ALT, AST, CK, and LDH and increased WBCs in CTX-induced mice. In addition, DBD significantly alleviated pathological changes of heart tissue. DBD significantly reduced the protein expressions of NF-кB signaling pathway. In summary, DBD can be considered an effective drug to alleviate CTX-induced heart damage in mice.

## 1. Introduction

Cardiovascular disease, a severe health problem, is recognized as one of the most burdensome diseases of society. Ischemia of the myocardium facilitates the development of arrhythmias which might further result in cardiac necrosis [1]. Cyclophosphamide (CTX), an alkylating agent, is a widely acknowledged anticancer chemotherapeutic agent used alone or in combination with other medicines for part of the mainstream therapy of several human malignancies [2]. Due to its immunosuppressive activity, CTX could also be used in preconditioning the host for immunotherapy. Emerging evidence elicited that the administration with CTX increased the numbers of the dendritic cells that directly drove the immune response and activation of immune-reaction by promoting the mobilization of the hematopoietic stem cells [3]. Despite many beneficial effects, several adverse side effects of CTX have been reported, such as heart toxicity with high incidence. Exposure to high dose CTX could attribute to the acute cardiotoxic effects characterized by myocyte damage, extravasation of toxic metabolites, and diastolic contractile dysfunction [4]. Herein, we employed the injection of CTX as an experimental model in our study.

NF-кB is a key transcriptional factor involved in inflammatory progression of various diseases [5–7]. Accumulating evidence indicated the pivotal role of NF-кB in cardiac dysfunction. It is well known that the IKK complex could mediate the phosphorylation and degradation of IкB, which ultimately activates NF-кB signaling pathway [8]. The activation of NF-кB would conduce to the productions of inflammatory cytokines and the modulations of other biochemical indices of heart disease such as LDH and CK [9].

Traditional Chinese medicine has been acknowledged as a major source of medicine, which was used for centuries around the world [10, 11]. Dangguibuxue decoction (DBD), a Chinese medicinal decoction that contains* Angelicae sinensis* radix (Danggui) and* Astragalus radix* (Huangqi) at a ratio of 1:5, has been widely used to treat blood deficiency disease in China for more than 800 years because of the hematopoietic properties of this prescription [12]. In Chinese, the herb Angelica sinensis is pronounced as Dang Gui; Bu Xue refers to hematopoietic effects. Recent findings suggested that DGBUT has the ability to promote hematopoietic function, inhibit platelet aggregation, and stimulate cardiovascular circulation [13]. Furthermore, the anti-inflammatory activity of Radix Astragali extract has been proved by substantial researches [14]. As to our knowledge, there are few available reports associated with the protective effect of DBD on CTX-induced alterations. Therefore, the present study was aimed at addressing the ameliorated effect and exploring the protective underlying mechanism of DBD on CTX-stimulated heart injury.

## 2. Materials and Methods

### 2.1. Chemicals and Regents

CTX was purchased from (Sigma-Aldrich, St. Louis, MO). ALT, AST, CK, and LDH assay kits were purchased from Nanjing Jiancheng Bioengineering Institute (Nanjing, China). All antibodies were supplied from Cell Signaling Technology Inc. (Beverly, MA, USA).

DBD consists of Radix Astragali and Angelica, with a ratio of 5:1. Herbal medicine was authenticated by pharmacists which was provided by Shanghai Huayu Chinese Medicine Co., Ltd., and was produced in Gansu Province. DBD is prepared by boiling water and alcohol extraction. The extract is then sprayed and dried to give residue. The dried residue dissolves in water and gives an oral solution of DBD at a specified concentration and remains at 4°C.

### 2.2. HPLC Analysis of DBD Decoction

HPLC was performed using Agilent 1200 HPLC with G 1321 A FD and Eclipse AAA columns (4.6× 150 mm, 5 m) and column temperature of 40°C. Mobile phase A (formic acid: water = 1: 1000) and mobile phase B (acetonitrile). Gradient elution procedure is 0-70 minutes, B (0%-63%), 0-20 minutes, B (63%), 20-40 minutes, B (100%), 50 minutes, B (100%-0%), and 50-70 minutes, B (0%). The flow rate was 0.4 ml min^−1^.

### 2.3. Animals and Treatment

The male ICR mice (20±2 g), acquired from Nantong University (Nantong, China), were maintained prior to experiments in an animal room under standard conditions (23±2°C temperature, 60±10% humidity, 12 h light/dark cycle).

40 male ICR mice were divided into 4 groups; mice in group 1 were given 200 mL of phosphate buffer saline (PBS, orally), mice in group 2 were given a single dose of CTX (200 mg/kg, intraperitoneally), mice in group 3 were given a single dose of CTX (200 mg/kg, intraperitoneally) and then were given DBD (6 g/kg, orally) for 7 consecutive days, and mice in group 4 were given a single dose of CTX (200 mg/kg, intraperitoneally) in the abdominal cavity and then were given DBD (12 g/kg, orally) for 7 consecutive days. Thereafter, after 4 days without administration, at day 12, the blood comes from the orbital plexus of the eye to obtain a serum for subsequent hematology or biochemical analysis. After collection of blood samples, all mice were sacrificed and their spleens were removed and weighed.

### 2.4. Hematological Determination

The total number of WBCs and platelets was detected in fresh blood samples from all mice using Automatic Biochemical Analyzer. WBCs counts were performed on 0, 2, 4, 8, and 12 day using Automatic Biochemical Analyzer.

### 2.5. Cytokine Measurement

For IL-6, TNF-*α*, and IL-1*β* in the heart, the mice heart samples were homogenized in the ice in an aqueous salt solution and then centrifuged at 2500 × g for 10 min at 4°C to obtain supernatants. According to the manufacturer's instructions, the IL-6, TNF-*α*, and IL-1*β* in the heart were measured by the Elisa kit. The protein content of these samples was detected by BCA test kit and normalized with cytokine parameter data.

### 2.6. Biochemical Determination

The levels of AST, ALT CK, and LDH in serum were assayed in consistence with the instruments of commercial test kits (Jiancheng Bioengineering Inc., China).

### 2.7. Histological Analysis of Myocardium

Mice were intraperitoneally injected with 20% urethane, were anesthetized according to their body weight (0.5 ml/100 g), cut the heart, cut off the excess tissue around the heart, washed the blood stains in normal saline, drained the excess water from the heart with filter paper, were fixed in 4% paraformaldehyde for 24 h, then were dipped in wax, were embedded, and prepared 4 *μ*M paraffin sections. Routine HE staining, xylene transparency, gradient alcohol dehydration, hematoxylin staining, hydrochloric acid differentiation, eosin staining, alcohol dehydration transparency, resin sealing, and the pathological morphology of myocardial tissue were observed under a 200-fold microscope.

### 2.8. Western Blot

RIPA lysate was used to extract the total protein in the heart tissue of each group of mice, centrifuged at 12,000 rpm for 15 min, and the supernatant was collected. The BCA kit was used to quantify each group of proteins. Each protein sample was subjected to SDS-polyacrylamide gel electrophoresis and then transferred to PVDF membrane. The PVDF membrane was then sealed in 5% skim milk for 2 h. After 2 hours of blocking, the PVDF membrane was placed in the corresponding primary antibody and incubated at 4°C overnight. The next day TBST was washed 4 times for 8 min each time. The PVDF membrane was then placed in a secondary antibody solution and incubated for 2 h at room temperature in a shaker. The PVDF membrane was removed and washed 4 times with TBST for 8 min each time. The gray value of each strip was analyzed by exposing and scanning the strip using a gel imaging system and using densitometry of Bandscan 5.0 software that was used for quantifying the density of each protein band.

### 2.9. Immunohistochemistry

Immunohistochemical method was used to detect the expression of p-NF-*κ*BP65 in the heart. In short, heart tissue was fixed with 4% paraformaldehyde (PFA), embedded in paraffin, and sliced. Paraffin sections were dewaxed in xylene and anhydrous ethanol, microwaved in sodium citrate buffer, and washed with PBS. Endogenous peroxidase activity was blocked by 3% hydrogen peroxide for 20 min. Each sample was sealed with 5% goat serum for 20 minutes, then treated with primary antibody p-NF-*κ*BP65 (1: 200, # 8242, cell signaling technique), and stayed at 4°C overnight. The next day, after washing three times with PBS, each sample was treated with goat anti-rabbit IgG antibody for 20 min, then the streptavidin working solution labeled with horseradish enzyme was incubated for 20 min and washed three times with PBS. Then it was dyed with 3-3′ diaminobenzidine (DAB) and then with hematoxylin. After dehydration and drying, the slices were fixed with neutral glue and observed under a microscope.

### 2.10. Quantitative Real-Time PCR Analysis

According to the manufacturer's instructions, the total RNA of the heart was extracted from the heart tissue using the TRIZOL reagent (Invitrogen, Life Technologies, CA, USA). The purity of RNA was measured with 2000 thermal science nanodrops (Massachusetts, USA). Next, RNA was transcribed into cDNA using a reverse transcriptase kit (Takara Biotech, Kyoto, Japan). Quantitative real-time PCR (qRT-PCR) analysis was performed using a ChamQ SYBR qPCR Master Mix (Vazyme Biotech Co., Ltd., Nanjing, China) with the CFX Manager software (Bio-Rad Laboratories Inc.). GAPDH was analyzed in each sample for standardized expression. The primers used in this study are listed in Table 1. The relative expression was analyzed by 2^−ΔΔCt^.

### 2.11. Statistical Analysis

All data were presented as mean values ± SDs. Differences between groups of different treatments were evaluated by ANOVA with Tukey multiple comparison test, with P-values of 0.05 or less considered as statistical significance.

## 3. Results

### 3.1. HPLC Analysis of DBD

**Figure 1:**
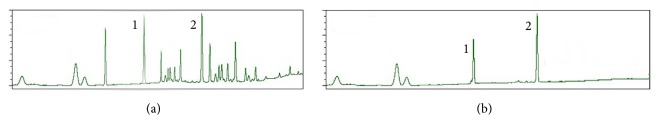
HPLC analysis of DBD. The main ingredients of DBD were detected by HPLC which was performed using Agilent 1200 HPLC with G1321A FD. (a) HPLC of DBD sample. (b) HPLC of standards. (1) Astragaloside IV. (2) Ferulic acid.

As shown in Figure 1, the contents of Astragaloside IV and Ferulic acid a have been identified as 0.112 microgram/mg and 0.878 microgram/mg, respectively.

### 3.2. Effect on the Weight of Spleen

**Figure 2:**
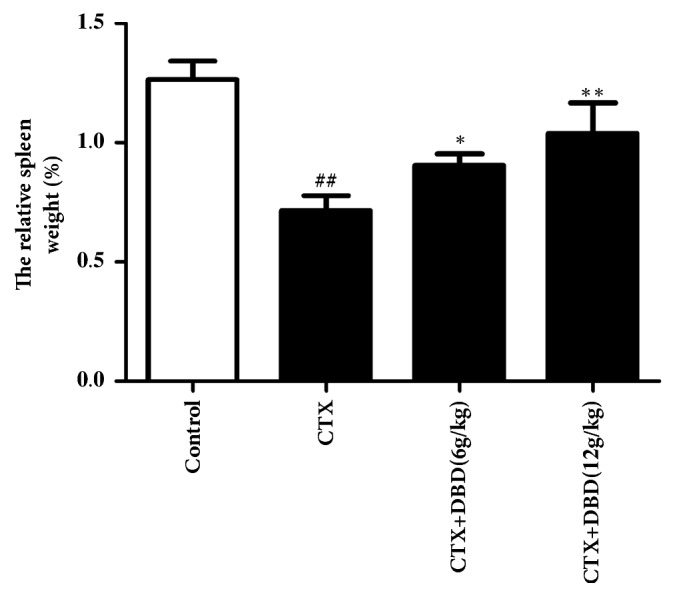
**Effects of DBD on weight of spleen.** At the end of experiment, mice were sacrificed and their spleens were removed and weighed.** All values given are the mean ± SD. **^#^**P<0.05 and **^##^**P<0.01 versus control group. ∗P<0.05 and ∗∗P<0.01 versus CTX- group.**

As it was shown in the data, there are significant changes in the spleen weights between the group 1 mice injected with PBS and other groups mice that were injected with CTX. Compared with the control group (group 1), the spleen weights of the mice that were injected alone with CTX shown decreased, while the mice injected with CTX along with two doses of DBD presented a marked elevation (p<0.05). Particularly, high doses DBD (12 g/kg) treatment facilitated the spleen weight growth, suggesting DBD seemed to exhibit its effect in conjunction with spleen function (Figure 2).

### 3.3. Effect on Hematological Parameters

#### 3.3.1. Total Number of WBCs

**Figure 3:**
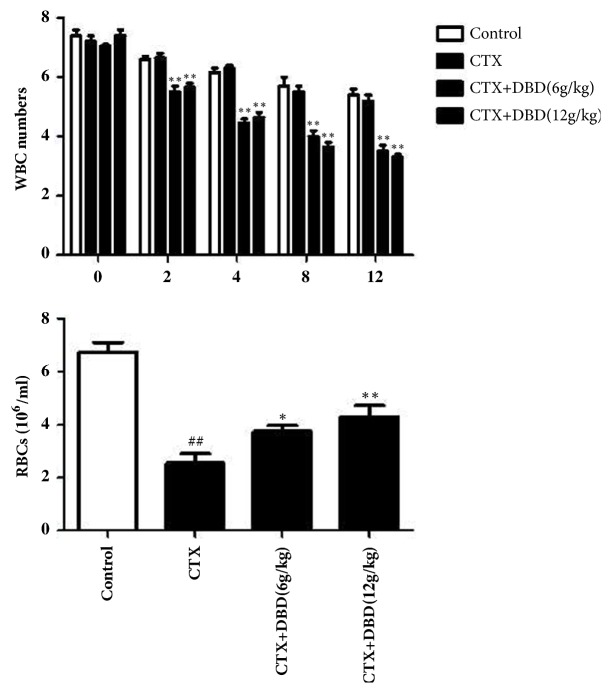
Effects of DND on hematological parameters. The white blood cell (WBCs) counts and the total platelets were detected from fresh blood samples acquired from the eyes of all groups with the electronic blood counter. All values given are the mean ± SD. ^#^P<0.05 and ^##^P<0.01 versus control group. *∗*P<0.05 and *∗∗*P<0.01 versus CTX- group.

Figure 3 suggested that a significant reduction (p<0.05) in the total number of WBCs presented between the normal group and CTX-injected groups including the alone CTX-injected group and DBD-treated groups until the 8th day. At the same time, there is a nonsignificant difference (p>0.05) in WBCs counts of two doses of CTX+DBD mice compared with that of the CTX alone mice. However, after 12 days, when compared with WBCs counts of the control group, the mice in the CTX alone group showed that their WBCs counts still kept a continuous tendency of going down, notably without the rapid reversion which were observed in the two DBD-treated groups.

#### 3.3.2. Total Number of Blood Platelets

In contrast to group 1, the mice injected alone with CTX in group 2 showed an observable decrease in the total number of the platelets. However, after DBD (6,12 g/kg) treatments animals in group 3 or 4 all increase directly the total number of the platelets closer to the level of normal group than that of model group (group 2). Interestingly, data suggested higher dose of DBD (12 g/kg) might obtain more apparent prevention in total number of blood platelets than a lower dose of DBD (6 g/kg) Figure 3.

### 3.4. Effect on Serum Biochemical Parameters

**Figure 4:**
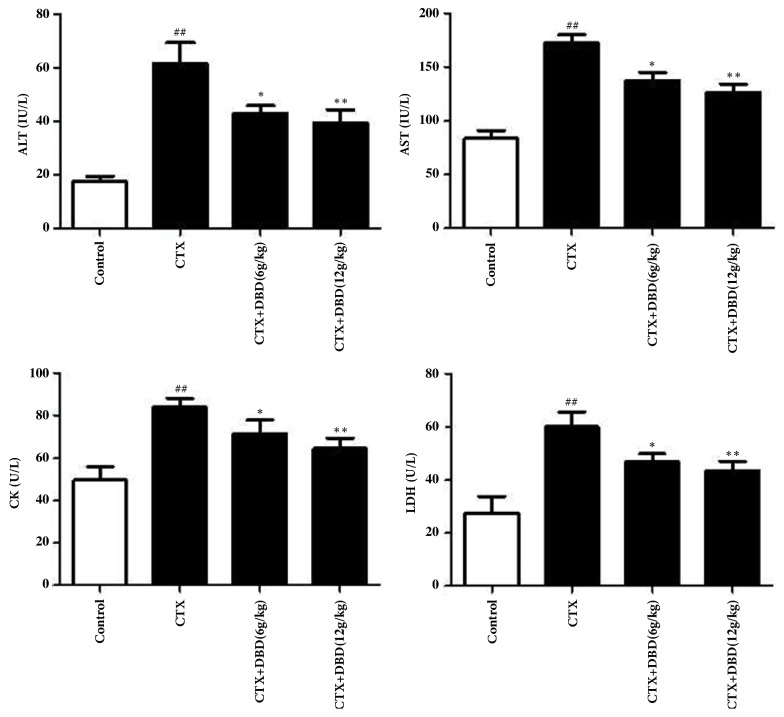
Effects of DND on serum biochemical parameters. Activities of serum aminotransferase including AST and ALT were assayed in consistence with the instruments of commercial test kits. LDH and CK levels in the serum were also measured according to the methods described by the protocols of commercially available standard kits. All values given are the mean ± SD. ^#^P<0.05 and ^##^P<0.01 versus control group. *∗*P<0.05 and *∗∗*P<0.01 versus CTX- group.

The serum levels of AST, ALT, CK, and LDH are features of myocardial damage. As depicted in Figure 4, the levels of these serum myocardial marker enzymes were markedly augmented in the model group (group 2) compared to those of the normal group (group 1), whereas DBD significantly reversed these increased levels to different degree, compared to the CTX-injected group (group 2). In addition, administration of DBD (12 g/kg) showed a more beneficial improvement in the levels of ALT, AST, CK, and LDH, with reverse such abnormal myocardial zymogram changes in serum to greater extent in comparison with those of DBD (6 g/kg).

### 3.5. Histopathological Examination of the Heart Tissues

**Figure 5:**
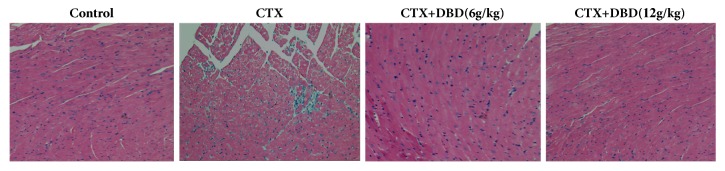
Histopathological examination of the heart tissues (x200). Tissues for histological analysis were formalin-fixed at room temperature and embedded in paraffin blocks were sliced into sections of 5 mm for being stained with (H&E).

Histological examination of the cardiac tissues from the control group (group 1) and model group (group 2) revealed a highly distinguishable architectural appearance. Histopathological examination of the cardiac tissues in control mice presented clear integrity of myocardial membrane and normal fibers without any infarction as well as infiltration of inflammatory cells. On the other hand, heart degeneration and infiltration of lymphocytes were observed in heart sections from CTX alone pretreatment, while these changes were mitigated in the DBD-treated groups, especially in the DBD (12 g/kg) group. Notably, treatment with DBD markedly reduced the numbers of inflammatory cells in infarcted region, indicating that DBD attenuated the histopathology condition of CTX-stimulated heart toxicity (Figure 5).

### 3.6. Effects of NF-кB Pathway in Heart Tissues

**Figure 6:**
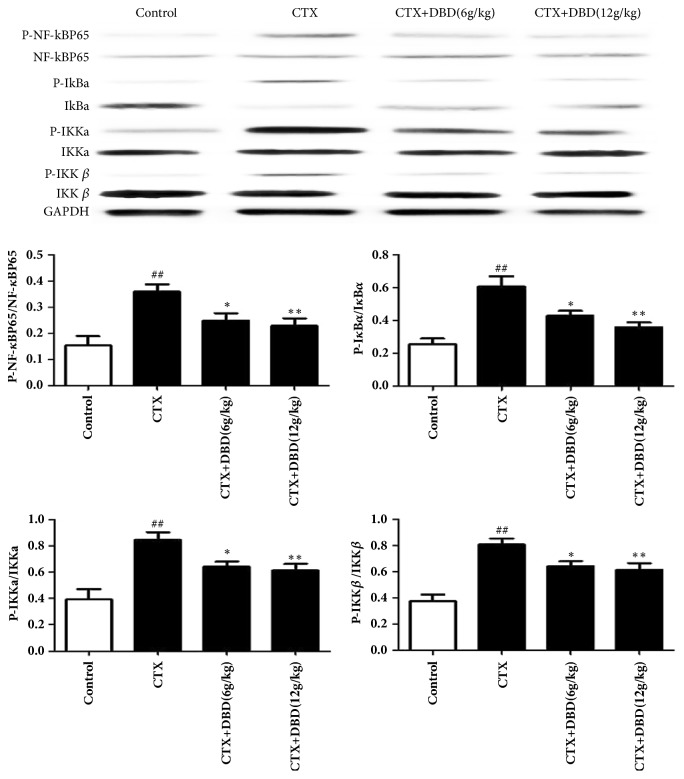
Effects of DND on NF-кB pathway in heart tissues. Western blot analysis was used to measure the NF-кB pathway in heart tissues. All values given are the mean ± SD. ^#^P<0.05 and ^##^P<0.01 versus control group. *∗*P<0.05 and *∗∗*P<0.01 versus CTX- group.

**Figure 7:**
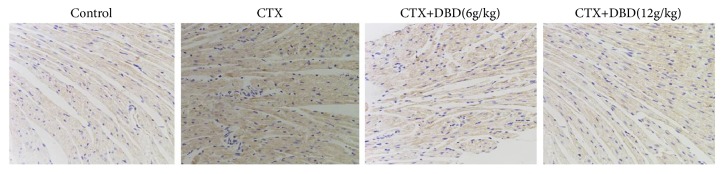
Effects of DND on p-NF-кBp65 in heart by immunohistochemical (x200). Immunohistochemical analysis was used to measure the p-NF-кBp65 in heart tissues.

To further assert the cardioprotective mechanism of DBD, the nonphosphorylated and phosphorylated forms of the NF-кB components were detected. Western blot analysis revealed that the exposure to CTX apparently upregulated the levels of p-NF-кBp65, p-IкB*α*, p-IKK*α*, and p-IKK*β* in contrast with those in control group. By contrast, treatment with DBD (6,12 g/kg) exhibited different degrees of downregulation of p-NF-кBp65, p-IкB*α*, p-IKK*α*, and p-IKK*β* in the alone CTX-pretreated mice. Our analytical results demonstrated that the inhibitory effects of phosphorylated NF-кB pathway caused by DBD might contribute to the ameliorative effect of Dangguibuxue decoction against cyclophosphamide-induced heart toxicity in mice (Figure 6). The results were consistent with the immunohistochemical result (Figure 7).

### 3.7. Effect of DBD on Cytokine in Heart

**Figure 8:**
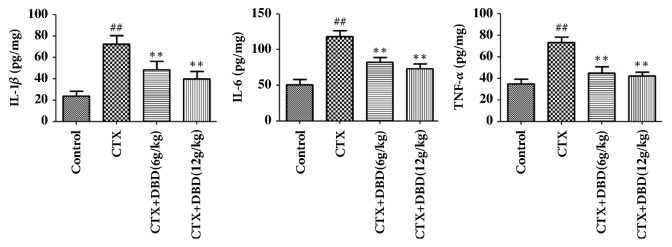
**Effect of DBD on cytokine in heart.** For IL-6, TNF-*α*, and IL-1*β* assay in heart, the mice heart samples were homogenized on ice in saline solution and then centrifuged at 2500 × g for 10 min at 4°C to get the supernatants. IL-6, IL-1*β*, and TNF-*α* levels in heart measured by ELISA kits.** All values given are the mean ± SD. **^#^**P<0.05 and **^##^**P<0.01 versus control group. ∗P<0.05 and ∗∗P<0.01 versus CTX- group.**

As depicted in Figure 8, IL-6, IL-1*β*, and TNF-*α* levels in heart were increased in CTX-induced mice. By contrast, treatment with DBD (6,12 g/kg) could reduce the levels of IL-6, IL-1*β*, and TNF-*α*in heart.

### 3.8. Effect of DBD on AST, ALT, CK, and LDH by PCR

**Figure 9:**
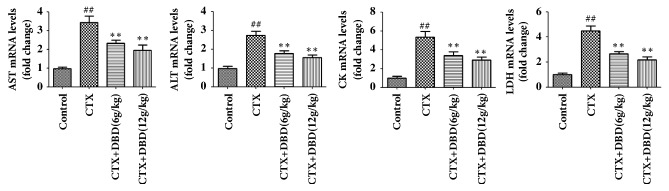
Effect of DBD on AST, ALT, CK, and LDH in heart by PCR. PCR analysis was used to measure the AST, ALT, CK, and LDH in heart tissues. All values given are the mean ± SD. ^#^P<0.05 and ^##^P<0.01 versus control group. *∗*P<0.05 and *∗∗*P<0.01 versus CTX- group.

As depicted in Figure 9, AST, ALT, CK, and LDH mRNA levels in heart were increased in CTX-induced mice. By contrast, treatment with DBD (6,12 g/kg) could reduce the AST, ALT, CK, and LDH mRNA levels in heart.

## 4. Discussion

Dangguibuxue decoction (DBD) containing Angelicae sinensis radix (Danggui) and Astragalus radix (Huangqi) at a ratio of 1:5 is characterized by anti-inflammation and antioxidantion, which can possibly exert a positive impact on the therapy method of cardiovascular diseases. This study was aimed at investigating the ameliorative effect of Dangguibuxue decoction (DBD) after treatment with the chemotherapeutic agent CTX. DBD significantly inhibited the serum levels of ALT, AST, CK, and LDH. In addition, DBD effectively decreased total numbers of WBCs and blood platelets and attenuated the histological change. Western blot analysis revealed that DBD ameliorated CTX-challenged heart injury possibly through the IKK/I*κ*B/NF-*κ*B signaling pathway.

The cyclophosphamide (CTX), a highly acknowledged alkylating agent, has been frequently applied for the intervention of neoplastic disease, such as leukemia or lymphomas [15]. There are studies indicating the fact that CTX exhibits the inhibitory effect against cancerous cells via promoting the proportion of suppressor cells in lymphoid organs and suppressing the function of the immune system [16]. However, the application of CTX could lead to heart toxicity contributing to the growth of morbidity and mortality [17]. Previous experiments have demonstrated the cardiac injury with the injection of CTX [18]. In the present work, we employed this model to examine the protective effect of Dangguibuxue decoction on CTX-induced heart injury.

It is well known that the spleen plays essential roles in regard to the immune system [19]. Spleen could remove old red blood cells by metabolizing hemoglobin which is removed from senescent erythrocytes. As a part of the mononuclear phagocyte system, the spleen can be regarded as a large lymph node, because its absence usually leads to a predisposition to infections [20]. Meanwhile, it has been reported in mice that half of the body's monocytes are reserved within the red pulp of spleen [21]. When tissue damage happened, these monocytes would move to injured tissue transforming into dendritic cells and macrophages to facilitate the treatment of tissue. In the present study, compared with the control group, there is an observable increase respectively in the weight of the spleen in the CTX and CTX along with DBD-injected groups, which suggested that the role of spleen might be involved in the protective effect of DBD against CTX-induced heart injury. Therefore, we conducted the hematological determination to confirm the hypothesis above.

The numbers of blood platelets, WBCs are the sensitive blood tests applied to the diagnosis the cardiac function in heart disease [22]. In CTX-challenged group, the enhanced hematological parameters were clearly evidenced via significant increases in the numbers of blood platelets, WBCs. By contrast, DBD provoked remission against CTX through the inhibitions of blood platelets, WBCs content. In consistent with the remarkable attenuation of histopathologic condition in heart tissue, these findings confirmed that DBD effectively ameliorated the heart injury caused by CTX treatment.

LDH is the specificity enzyme in the cytoplasm and releases into blood during myocardial dysfunction [23]. In addition, CK that distributes in the myocardium is widely treated as the contributing factors for heart damage [24]. As the common critical transaminases, AST and ALT are proved to mediate the cardiac metabolism [25]. Meanwhile, it was reported that the injection with CTX remarkably enhanced the toxicity in hearts of mice evidenced by the upregulations of LDH, CK, AST, and ALT [26]. The present study elucidated these phenomena and further revealed that DBD could ameliorate the lesion of heart toxicity through the suppression of LDH, CK, AST, and ALT.

Hence, we examined the hypothesis that treatment with DBD reduced the production of IL-1*β*, IL-6, and TNF-*α*. In general, IL-1*β* is involved in the repairment of acute inflammatory disorder. IL-6 contributes to expanding the inflammatory cascade in the pathogenesis of inflammation. TNF-*α* functions as a key factor in stimulating the generation of other inflammatory mediators and motivating innate immune response [27]. As expected, treatment with DBD (6, 12 g/kg) markedly suppressed the overproductions of proinflammatory cytokines triggered by CTX-challenged heart injury.

To further verify the property of the inhibitory effect of DBD on the secretion of inflammatory cytokine, we investigated the effects of DBD on the recruitment of the IKK/I*κ*B/NF-*κ*B pathways. As to our knowledge, it is highly acknowledged that NF-*κ*B signaling pathway is essential to the upregulation of inflammatory cytokines [28]. IKK complex consists of IKK-*α* and IKK-*β*, which are both the primary regulators participating in the NF-*κ*B pathway [29]. Consequently, the suppression of phosphorylated IKK*α*/*β* would contribute to the phosphorylation and degradation of I*κ*B-*α*, followed by the provocation of NF-*κ*B [30, 31]. Simultaneously, accumulating evidence indicated the upregulation of NF-кB activity has been also proved to mediate oxidative stress and inflammatory condition in myocardial ischemia mice [32, 33]. It was also demonstrated that the activation of NF-*κ*B was highly related to the pathogenesis of myocardial ischemia/reperfusion injury [34]. Thus, NF-*κ*B was designed to be a major target for the observation of CTX-induced cardiotoxic response with DBD treatment. As the result displayed, our analytical results revealed that DBD could exert the ameliorative effect against cyclophosphamide-induced heart injury in mice via the phosphorylations of IKK/IкB/NF-кB signaling pathway.

## 5. Conclusion

In conclusion, the present study demonstrated that the DBD administration apparently improved cardiac function after CTX-challenged heart toxicity in mice, suggesting a potential therapeutic role of DBD for the treatment of cardiovascular disorder. The cardioprotective effect of DBD might be attributed to its ability of suppressing biochemical indicators, which possibly partially occurred via the inhibition of the IKK/IкB/NF-кB pathway. Further studies are warranted to explore the clinical application of DBD in the future.

## Figures and Tables

**Table 1:** Primers used in this study.

Genes	GenBank	Primer sequence (5′-3′)	Primer sequence (3′-5′)
GAPDH	NM_018007	CTGAGGACCAGGTTGTCTCC	GAGGGCCTCTCTCTTGCTCT
AST	NM_018172	TCAATATGGGGACAATACAC	TACTTTCTTCATTTCCACCTT
ALT	NM_018109	TGTATGAAAGTGCTCAAGAT	GCCCTCTTGTGAGTATAAGT
CK	NM_018081	CGAACTACTTTATGCCC	GAAGACAAACGAGGTCTCTA
LDH	NM_001890	TATCGAGTCGAGTACGCCAA	GTGTGGGACTTTTCCATCAAA

## Data Availability

The data used to support the findings of this study are available from the corresponding author upon request.
